# hnRNP A1 antagonizes cellular senescence and senescence‐associated secretory phenotype via regulation of SIRT1 mRNA stability

**DOI:** 10.1111/acel.12511

**Published:** 2016-09-09

**Authors:** Hui Wang, Limin Han, Ganye Zhao, Hong Shen, Pengfeng Wang, Zhaomeng Sun, Chenzhong Xu, Yuanyuan Su, Guodong Li, Tanjun Tong, Jun Chen

**Affiliations:** ^1^ Peking University Research Center on Aging Department of Biochemistry and Molecular Biology Peking University Health Science Center Beijing China

**Keywords:** hnRNP A1, IL‐6, NF‐κB acetylation, RNA‐binding protein, senescence‐associated secretory phenotype, SIRT1

## Abstract

Senescent cells display a senescence‐associated secretory phenotype (SASP) which contributes to tumor suppression, aging, and cancer. However, the underlying mechanisms for SASP regulation are not fully elucidated. SIRT1, a nicotinamide adenosine dinucleotide‐dependent deacetylase, plays multiple roles in metabolism, inflammatory response, and longevity, etc. However, its posttranscriptional regulation and its roles in cellular senescence and SASP regulation are still elusive. Here, we identify the RNA‐binding protein hnRNP A1 as a posttranscriptional regulator of SIRT1, as well as cell senescence and SASP regulator. hnRNP A1 directly interacts with the 3′ untranslated region of SIRT1 mRNA, promotes its stability, and increases SIRT1 expression. hnRNP A1 delays replicative cellular senescence and prevents from Ras OIS via upregulation of SIRT1 expression to deacetylate NF‐κB, thus blunting its transcriptional activity and subsequent IL‐6/IL‐8 induction. hnRNP A1 overexpression promotes cell transformation and tumorigenesis in a SIRT1‐dependent manner. Together, our findings unveil a novel posttranscriptional regulation of SIRT1 by hnRNP A1 and uncover a critical role of hnRNP A1‐SIRT1–NF‐κB pathway in regulating cellular senescence and SASP expression.

## Introduction

Cellular senescence, including oncogene‐induced cellular senescence (OIS), is a stable cell cycle arrest which limits the proliferation of damaged cells and acts as a natural barrier against tumor development *in vivo* (Collado & Serrano, 2010). One distinct feature of senescent cells compared with young cells is that senescent cells secret a wide range of cytokines, chemokines, and other proteins termed as senescence‐associated secretory phenotype (SASP). SASP plays multiple biological functions such as tumor suppression, tissue repair, and embryonic development, by either autocrine or paracrine fashion. However, with senescent cell accumulation in late life, SASP can promote tumor formation and invasion and may contribute to aging and many age‐related diseases (van Deursen, 2014; Salama *et al*., 2014). Many SASP factors including key components IL‐6 and IL‐8 are mainly regulated at transcriptional level by transcription factor NF‐κB (Acosta *et al*., 2008; Kuilman *et al*., 2008). Despite various signaling pathways participate in regulating SASP production by altering NF‐κB pathway components phosphorylation status and activities (Wang *et al*., 2014; Kang *et al*., 2015), little is known about whether NF‐κB itself and its activity are subjected to other posttranslational modifications and regulations during senescence. Previous report shows that acetylation of lysine 310 in p65/RelA is required for the full transcriptional activity of NF‐κB (Chen *et al*., 2002). However, whether the acetylation status of NF‐κB is altered during senescence, whether NF‐κB acetylation has effect on the SASP expression, and how NF‐κB acetylation is regulated during senescence remain largely unknown.

SIRT1, an NAD‐dependent deacetylase, implicates in improving metabolism and preventing from various age‐related diseases to promote health span in mammals by deacetylating diverse targets such as histones H1, H3, and H4, or a large number of nonhistone substrates including p53, forkhead proteins, etc. (Luo *et al*., 2001; Vaziri *et al*., 2001; Brunet *et al*., 2004; Motta *et al*., 2004; Vaquero *et al*., 2004; Yeung *et al*., 2004; Deng, 2009). SIRT1 can protect against endothelial cell senescence via deacetylation of LKB1 to counteract LKB1‐AMPK activation‐provoked endothelial cell senescence (Zu *et al*., 2010). SIRT1 also suppresses SASP factors IL‐6/IL‐8 expression by deacetylating histones H3 (K9) and H4 (K16) of the IL‐6/IL‐8 promoter regions (Hayakawa *et al*., 2015). Moreover, SIRT1 is able to deacetylate p65 at lysine 310 to inhibit transcriptional activity of NF‐κB (Yeung *et al*., 2004). However, whether SIRT1 can regulate SASP induction by altering NF‐κB acetylation level is still unknown.

SIRT1 itself also undergoes variety of modifications at different levels. SIRT1 is subjected to sumoylation modification on Lys 734 which increases its intrinsic activity **(**Yang *et al*., 2007 **)**. The RNA‐binding protein HuR associates with the 3′‐untranslated region (3′UTR) of SIRT1 mRNA to stabilize its mRNA, thus increasing SIRT1 expression (Abdelmohsen *et al*., 2007). However, the upstream cues which can regulate SIRT1 expression and function at posttranscriptional level are still poorly defined.

Heterogeneous nuclear ribonucleoprotein A1 (hnRNP A1) belongs to a large family of hnRNPs termed A–U which play important roles in mRNA metabolism such as alternative splicing and mRNA stability (Dreyfuss *et al*., 2002; He & Smith, 2009). hnRNP A1 also implicates in telomere length regulation and telomere protection (LaBranche *et al*., 1998; Ding *et al*., 1999). Although hnRNP A1 level decreases in senescent fibroblasts and hnRNP A1 depletion induces senescence phenotype (Shimada *et al*., 2009), however, the underlying mechanism is largely unknown and no direct evidence to support that hnRNP A1 regulate cellular senescence dependent on its telomere maintenance function. hnRNP A1 has been shown to regulate UV‐induced NF‐κB signaling through destabilization of cIAP1 mRNA (Zhao *et al*., 2009). However, whether hnRNP A1 can regulate cellular senescence and SASP expression by modulating NF‐κB activity needs to be explored.

In this study, we identify hnRNP A1 as a novel SIRT1 regulator which can bind to the SIRT1 mRNA 3′UTR and stabilize its mRNA, thus elevating SIRT1 expression at posttranscriptional level. We further demonstrate that hnRNP A1 can suppress cellular senescence and SASP induction, which depends on SIRT1‐mediated deacetylation of NF‐κB.

## Results

### hnRNP A1 directly associates with SIRT1 mRNA

**Figure 1:**
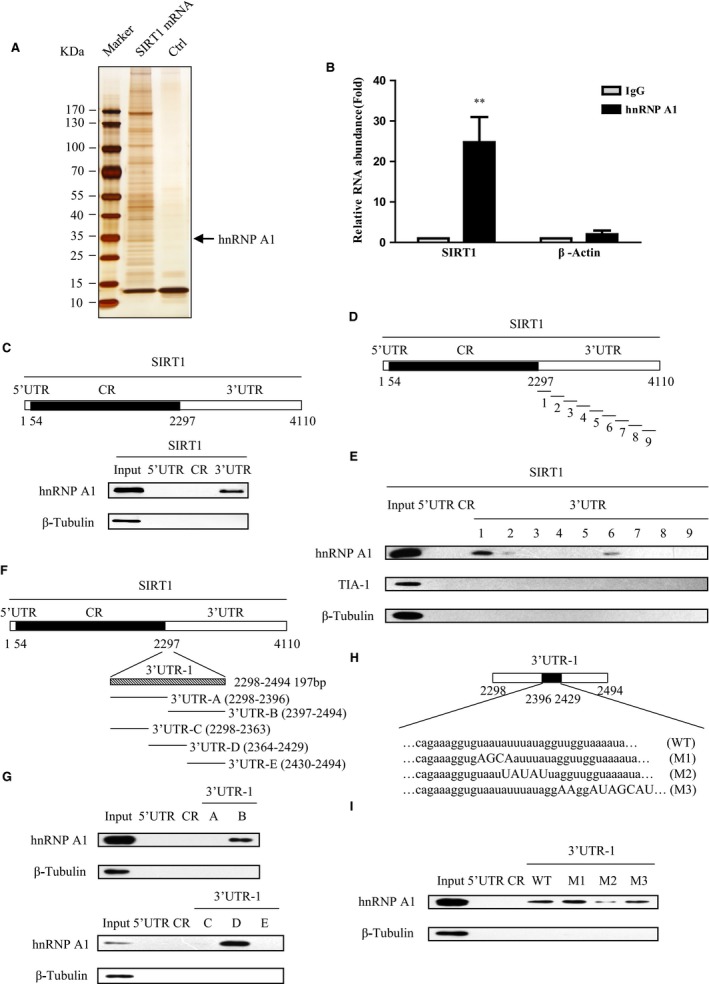
hnRNP A1 interacts with SIRT1 mRNA. (A) hnRNP A1 is pulled down by SIRT1 mRNA. 2BS cell extract incubated with streptavidin‐coupled beads (control) or with biotin‐labeled SIRT1 mRNA and beads. The elutes were separated by SDS‐PAGE and silver‐stained. The protein bands were retrieved and analyzed by mass spectrometry. The arrow points to the hnRNP A1 signal. (B) SIRT1 mRNA is immunoprecipitated by hnRNP A1 antibody. Total RNA from HeLa cell subjected to RNP‐IP assays using hnRNP A1 and IgG antibody. Real‐time qPCR analyzed the immunoprecipitated mRNA. Actin mRNA was included as a negative control. Error bars represent means + S.D. (n=3) ** *P* <0.01. (C) hnRNP A1 binds to SIRT1 mRNA 3′UTR. Upper panel, schematic representation of SIRT1 mRNA 5′UTR, CR, and 3′UTR. Bottom panel, biotinylated fragments of 5′UTR, CR, and 3′UTR were subjected to biotin pull‐down assay to detect bound hnRNP A1 by Western blot. β‐Tubulin which was not a RNA‐binding protein served as a negative control. (D–I) Identify the specific binding region of hnRNP A1 to SIRT1 mRNA 3′UTR. (D) Schematic diagram of SIRT1 mRNA, and 9 different fragments of SIRT1 mRNA 3′UTR. (E) Biotinylated RNA fragments were subjected to RNA pull‐down assay to detect bound proteins by Western blot. RNA‐binding protein TIA‐1 served as a negative control. (F) The graphic depiction of SIRT1 mRNA, and 2 or 3 fragments of SIRT1 mRNA 3′UTR‐1. (G) Biotinylated RNA fragments were subjected to RNA pull‐down assay following Western blot. SIRT1 mRNA 5′UTR and CR were considered as negative RNA probes to hnRNP A1. (H) Schematic diagram of wild‐type 3′UTR‐1 and three mutants M1, M2, and M3. The mutation sites are marked by capital letters. (I) AUUUA pentamer in SIRT1 mRNA 3′UTR is essential for the association of hnRNP A1 with SIRT1 3′UTR. Biotinylated fragments WT, M1, M2, and M3 were subjected to RNA pull‐down assay following Western blot. In all biotinylated RNA pull‐down assays, 5 μg aliquot of whole‐cell lysate was included as input, and β‐tubulin served as a negative control.

In view of the lacking of the knowledge for posttranscriptional regulation of SIRT1, we first employed RNA pull‐down assay and mass spectrometry to interrogate SIRT1 mRNA‐binding proteins *in vivo*. Full‐length biotinylated transcript of SIRT1 mRNA and total cell lysates were incubated and pulled down by streptavidin‐conjugated magnetic beads. Western blot analysis using a gradient gel and silver staining was performed. Mass spectrometry analysis indicated that hnRNP A1, a RNA‐binding protein, was in the presence of the SIRT1 mRNA interactome (Fig. 1A). The detailed results of mass spectrometric analysis are provided in Table S1. HuR, a previous identified SIRT1 mRNA‐binding protein (Abdelmohsen *et al*., 2007), was also on the list, proving the validity and rationality of our methodology.

To further prove the interaction between SIRT1 mRNA and hnRNP A1, we carried out RNP‐IP assay by incubating anti‐hnRNP A1 antibody with total RNA extracts from HeLa cells. The potential RNA‐hnRNP A1 binding complexes were eluted and analyzed by real‐time PCR specific for SIRT1 mRNA. As shown in Fig. 1B, the SIRT1 mRNA was significantly enriched in hnRNP A1‐IP sample compared with negative control IgG‐IP sample. Negligible binding of β‐actin transcript with hnRNP A1 demonstrated that the interaction between SIRT1 mRNA and hnRNP A1 was specific.

To delineate the specific binding region of SIRT1 mRNA to hnRNP A1, we amplified 5′UTR, coding region (CR), and 3′UTR of SIRT1 mRNA labeled by biotin *in vitro*. Then, the biotin pull‐down assay was executed followed by Western blot analysis. The result indicated that hnRNP A1 interacted with the 3′UTR of SIRT1 mRNA, instead of 5′UTR or CR of SIRT1 mRNA (Fig. 1C).

To further narrow down the specific binding region between hnRNP A1 and 3′UTR of SIRT1 mRNA, we split the full length of SIRT1 3′UTR into nine small fragments (Fig. 1D) and carried out the biotin pull‐down assay. The results indicated that fragment 3′UTR‐1 (positions from 2298 to 2494) was the main binding target of hnRNP A1 instead of fragments 3′UTR‐2 to 9, although 3′UTR‐2 and 3′UTR‐6 showed a weak binding with hnRNP A1 (Fig. 1E, and Fig. S1). No any signal was detected when incubation these nine fragments with RNA‐binding protein TIA‐1, which served as a negative control. Then, we separated 3′UTR‐1 into two parts, namely 3′UTR‐A and 3′UTR‐B, or three parts, namely 3′UTR‐C, 3′UTR‐D, and 3′UTR‐E, respectively, to further address the binding motif of SIRT1 3′UTR with hnRNP A1 (Fig. 1F). As shown in Fig. 1G, hnRNP A1 interacted with SIRT1 3′UTR‐B and 3′UTR‐D, respectively. These two fragments shared an overlapping region from positions 2397 to 2429. Moreover, the overlapping region contained an AUUUA pentamer motif which was frequently found in hnRNP A1 binding mRNAs (Hamilton *et al*., 1993, 1997). To test whether the AUUUA motif was essential for SIRT1 mRNA interaction with hnRNP A1, several 3′UTR‐1 mutants which mutated at flank regions of AUUUA motif or within this motif were generated (Fig. 1H), and followed by biotin RNA pull‐down assay. The M2 mutant wherein AUUUA was replaced by UAUAU drastically lost the ability to associate with hnRNP A1 compared with wild‐type fragment 3′UTR‐1. Conversely, mutants M1 and M3 interact with hnRNP A1 similar to wild‐type 3′UTR‐1 (Fig. 1I). Taken together, these results implicate that hnRNP A1 is the SIRT1 mRNA‐binding protein and hnRNP A1 directly binds to the SIRT1 mRNA 3′UTR, and AUUUA motif in SIRT1 3′UTR‐1 is essential for hnRNP A1 interaction with SIRT1 mRNA.

### hnRNP A1 upregulates SIRT1 expression by stabilizing its mRNA

**Figure 2:**
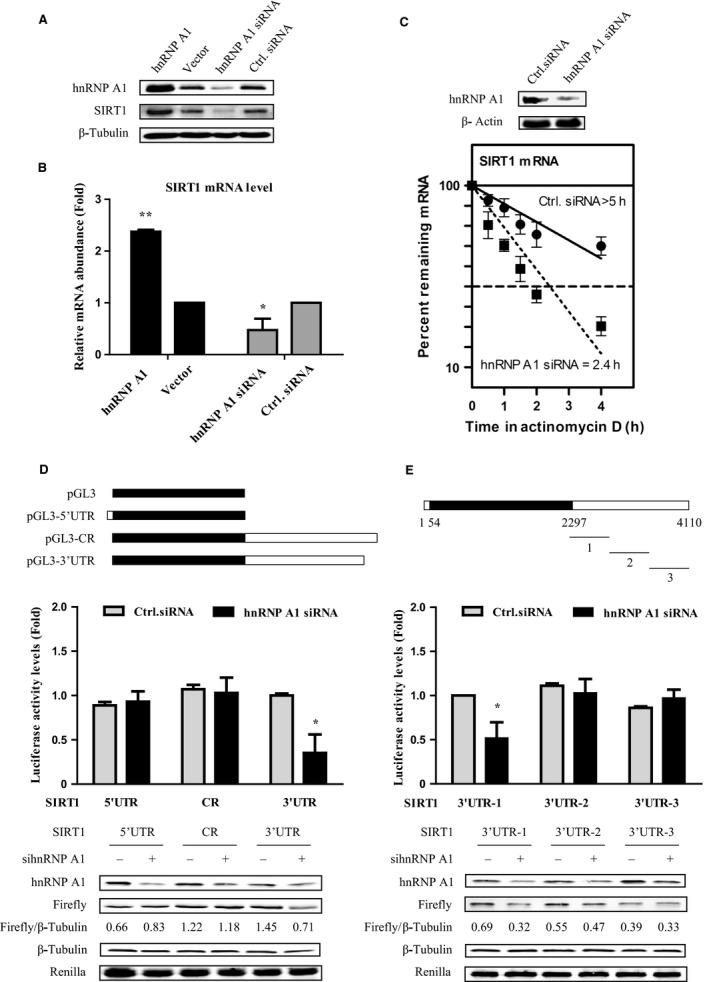
hnRNP A1 upregulates SIRT1 expression by stabilizing its mRNA. (A, B) hnRNP A1 overexpression enhances SIRT1, whereas hnRNP A1 silencing decreases SIRT1 expression. H1299 cells were transfected with hnRNP A1‐pcDNA3.1 and pcDNA3.1 vector. Forty‐four h later, whole‐cell lysates and total RNA were subjected to SDS‐PAGE or RT‐qPCR analysis. β‐Tubulin served as a loading control. (C) hnRNP A1 knockdown reduces SIRT1 mRNA half‐life. Cells were transfected with hnRNP A1 siRNA or control siRNA for 48 h and then exposed to actinomycin D (2 μg/ml), and total RNAs were isolated at indicated times and subjected to real‐time PCR to assess the half‐life of SIRT1 mRNA. (D, E) hnRNP A1 regulates SIRT1 depending on the interaction between hnRNP A1 and SIRT1 mRNA. pGL3‐derived luciferase reporter vectors bearing 5′UTR, CR, 3′UTR and three different fragments of SIRT1 3′UTR were cotransfected with Renilla vector to H1299 cells, respectively. Forty‐eight h later, cell lysates were collected, and the luciferase activities against Renilla luciferase activities were measured by the double‐luciferase assay system. Error bars represent as means ± SD from three independent experiments. *P <0.05, ** *P* <0.01.

Given that hnRNP A1 is the SIRT1 mRNA‐binding protein, next we explored what effect hnRNP A1 might have on the SIRT1. hnRNP A1 overexpression significantly enhanced SIRT1 expression at both translational and transcriptional levels, while hnRNP A1 silencing dramatically reduced SIRT1 protein and mRNA levels, compared with corresponding control cells (Fig. 2A,B, and Fig. S2). hnRNP A1 may enhance SIRT1 pre‐RNA level or promote SIRT1 mRNA stability to increase SIRT1 mRNA abundance. To test this, SIRT1 mRNA half‐life and SIRT1 pre‐RNA level in cells transfected with hnRNP A1 siRNA or control siRNA were assessed. hnRNP A1 knockdown markedly shortened SIRT1 mRNA half‐life (˜2.4 h) compared with mock siRNA (> 5 h) (Fig. 2C), whereas SIRT1 pre‐RNA level did not change with or without hnRNP A1 (Fig. S3). This result supports the view that hnRNP A1 stabilizes SIRT1 mRNA upon binding to its 3′UTR.

To investigate whether hnRNP A1 promotes SIRT1 expression depending on its binding capacity to SIRT1 mRNA, we constructed luciferase reporter plasmids containing SIRT1 5′UTR, CR, and 3′UTR, respectively, and cotransfected with hnRNP A1 siRNA. Deletion of hnRNP A1 only reduced the luciferase activity of pGL3‐3′UTR but not of pGL3‐5′UTR and pGL3‐CR (Fig. 2D). Then we split SIRT1 3′UTR into three parts and generated their corresponding luciferase reporter plasmids. Only SIRT1 3′UTR‐1 which binds with hnRNP A1 (Fig. S4) sharply lost the luciferase activity by silencing hnRNP A1, whereas the expression of 3′UTR‐2 and 3′UTR‐3 remained unchanged in the presence or absence of hnRNP A1 (Fig. 2E). These results indicate that hnRNP A1 increases SIRT1 expression that relies on its binding capability to SIRT1 mRNA.

### hnRNP A1 delays replicative cellular senescence in a SIRT1‐dependent manner

**Figure 3:**
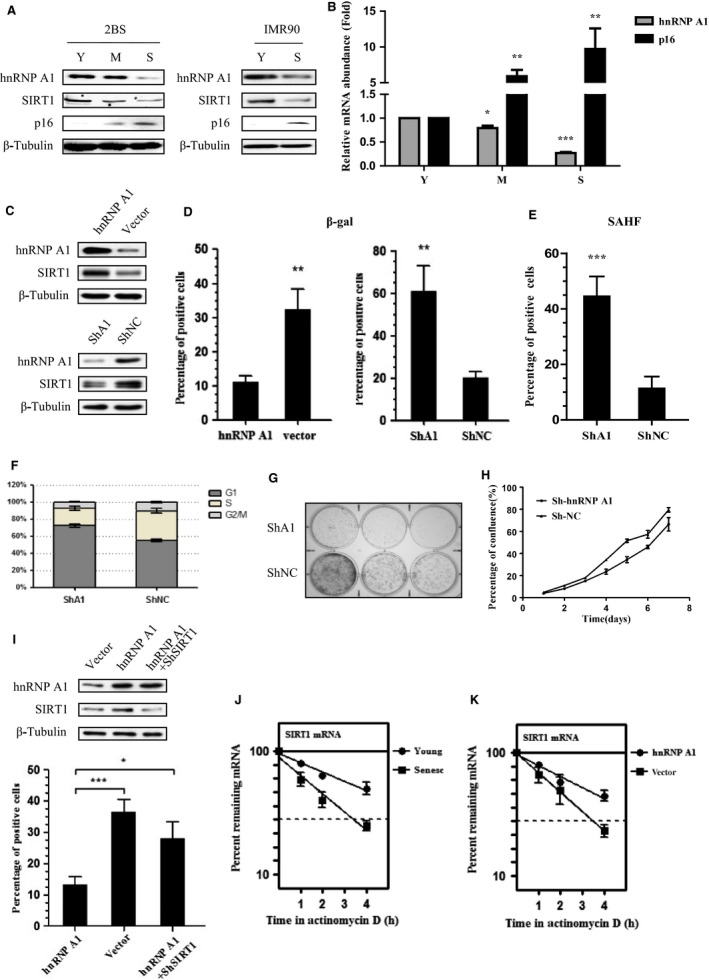
hnRNP A1 delays replicative cellular senescence dependent on SIRT1 in 2BS cells. (A, B) hnRNP A1 decreases in old cells. Total cell lysates and RNAs were extracted from young (Y), middle‐aged (M), and senescent (S) 2BS and IMR90 cells, and subjected to (A) immunoblotting for indicated antibodies, or (B) real‐time PCR analysis of hnRNP A1 mRNA levels. β‐Actin and β‐tubulin served as loading controls. Error bars represent mean ± SD for three independent experiments. (C–H) hnRNP A1 overexpression delays, while hnRNP A1 deletion accelerates replicative cell senescence. All stable cells were continuously passaged until the differences of senescence‐associated phenotypes between each stable cells displayed, and then, (C) cells are subjected to Western blot analysis. (D) Cells were stained for SA‐β‐gal. (E) Cells were stained for DAPI to detect SAHF formation. (F) Cell cycle was analyzed by flow cytometry. (G) Colony formation assay was performed. (H) Cell growth curves were determined by MTT assay. Values represent the means ± S.D. of triplicate points from a representative experiment (n = 3), which was repeated three times with similar results. (I) hnRNP A1 regulates cell senescence depending on SIRT1. Each stable cell was subjected to SA‐β‐gal staining after continuous passage to senescence phenotype displayed. (J) The half‐life of SIRT1 mRNA is shortened in old cells. The SIRT1 mRNA half‐life of young (28 PD) and senescent (51 PD) 2BS cells was assessed. (K) hnRNP A1 prolongs SIRT1 mRNA half‐life of senescent cells. Middle‐aged 2BS cells stably transfected with hnRNP A1 or control vector were cultured consistent to senescence, and then the half‐life of SIRT1 mRNA were assessed. Error bars represent as means ± SD from three independent experiments. *P <0.05, ** *P* <0.01, *** *P* <0.005.

Considering the role of SIRT1 in cell senescence and SASP regulation, and in light of the ability of hnRNP A1 to regulate SIRT1 expression, we speculated whether hnRNP A1 could modulate cellular senescence via regulation of SIRT1. We used human lung fibroblast strain 2BS cells as a replicative senescence model, and IMR90 cells stably expressing 4‐hydroxytamoxifen (4‐OHT)‐inducible ER:Ras fusion protein (ER:Ras‐IMR90 cells) as a Ras OIS model (Young *et al*., 2009). First, we evaluated hnRNP A1 expression levels in young, middle‐aged, and senescent 2BS and Ras‐induced senescent IMR90 cells. In agreement with previous report (Shimada *et al*., 2009), hnRNP A1 expression gradually decreased to barely detectable level during cellular senescence. SIRT1 had similar expression change pattern with hnRNP A1, whereas p16 INK4a level increased substantially following cellular senescence (Fig. 3A). In addition, hnRNP A1 mRNA level also progressively reduced during senescence in contrast to the steady increase in p16 INK4a mRNA level (Fig. 3B).

To determine the functional role of hnRNP A1 in cellular senescence, we stably overexpressed or silenced hnRNP A1 in young 2BS cells and examined their effects on the replicative cellular senescence (Fig. 3C, Fig. S5, and Fig. S7A, 7B). hnRNP A1‐overexpressed cells showed low SA‐β‐gal staining (Fig. 3D, and Fig. S6A), compared with corresponding control cells. In contrast, depletion of hnRNP A1 induced robust senescence phenotypes compared with corresponding mock cells, which included elevated SA‐β‐gal activity (Fig. 3D, Fig. S6A, and Fig. S7C), far more senescence‐associated heterochromatin foci (SAHF) (Fig. 3E, and Fig. S6B), reduced S phase, and increased G1 compartment which indicate G_0_/G_1_ cell cycle arrest (Fig. 3F), much less colonies (Fig. 3G), and cell growth retardation (Fig. 3H). These results reveal that hnRNP A1 plays an important role in cellular senescence, hnRNP A1 overexpression promotes cell proliferation and delays replicative cellular senescence, whereas hnRNP A1 ablation leads to premature senescence, which agrees with previous data (Shimada *et al*., 2009).

To exploit whether hnRNP A1 depends on SIRT1 to regulate cellular senescence, we cotransfected hnRNP A1 with SIRT1‐shRNA in 2BS cells and assessed senescence response (Fig. 3I, Fig. S6C, and Fig. S7D). The decrease in SA‐β‐gal activity in hnRNP A1‐overexpressed cells was remarkably lost when SIRT1 was deleted (Fig. 3I, Fig. S6D, and Fig. S7E). We also observed that neither overexpression nor depletion of hnRNP A1 altered p53/p21, p16, and PTEN/p27 level (Fig. S6E). These results suggest that hnRNP A1 postpones replicative senescence at least partially via SIRT1.

We further determined the half‐life of SIRT1 mRNA in young and senescent 2BS cells. The SIRT1 mRNA half‐life in young cells (28 PD) was much longer than it in senescent cells (51 PD) (Fig. 3J). In contrast, ectopic expression of hnRNP A1 rescued the decline in half‐life of SIRT1 mRNA in senescent cells (Fig. 3K), which implied that hnRNP A1 promoted SIRT1 expression to defer replicative senescence via increasing SIRT1 mRNA stability. Together, these data indicate that hnRNP A1 regulates replicative cellular senescence in a SIRT1‐dependent manner.

We also analyzed hnRNP A1 and SIRT1 expressions in several tissues from young and old BALB/c mice. The overall levels of hnRNP A1 and SIRT1 in aged mice significantly diminished in liver, fat, and heart, and mildly declined in muscle, when compared with young mice (Fig. S8A‐8D). These results exhibit that hnRNP A1 and SIRT1 levels decrease with aging in both cells and tissues.

### SIRT1 inhibits NF‐κB acetylation and SASP induction in Ras OIS

**Figure 4:**
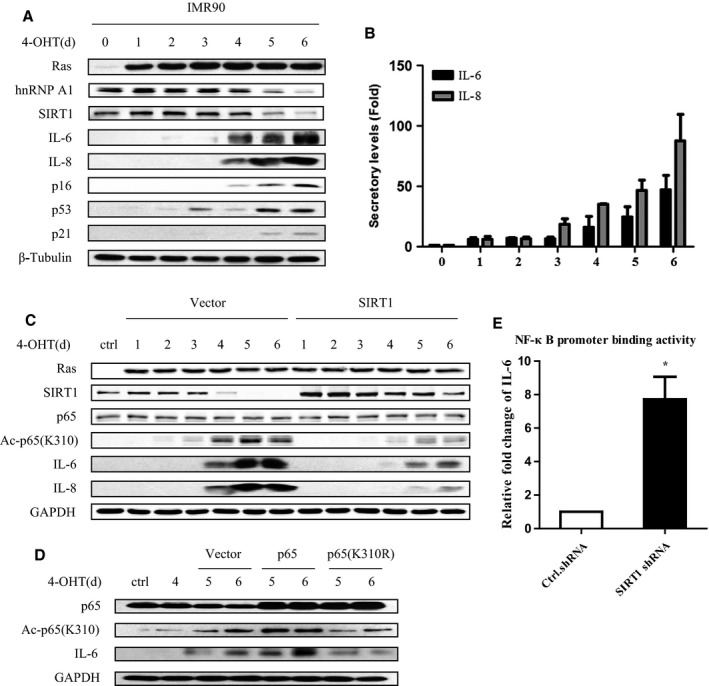
SIRT1 suppresses RelA/p65 acetylation on K310 and IL‐6/IL‐8 expression in Ras OIS. (A, B) SIRT1 and hnRNP A1 levels declined during Ras OIS. ER:Ras IMR90 cells were given 100 nM 4‐OHT for the indicated days, and fresh medium with 4‐OHT was changed every other day. Cell lysates and supernatants were collected every day for total 6 days, and then, (A) cell lysates were subjected to Western blot analysis for the indicated proteins, and (B) supernatants were analyzed secretory levels of IL‐6/IL‐8 by ELISA. (C) SIRT1 overexpression inhibits p65 acetylation and IL‐6/IL‐8 induction. ER:Ras IMR90 cells stably transfected with SIRT1 or control vector were exposed to 4‐OHT for the indicated days. Cell lysates were then subjected to Western blot for the indicated proteins. (D) p65 acetylation is essential for IL‐6/IL‐8 induction. ER:Ras IMR90 cells stably transfected with p65 or p65 (K310R) were exposed to 4‐OHT for the indicated days, and then, cell lysates were subjected to Western blot analysis. (E) Deletion of SIRT1 enhances NF‐κB binding activity to the IL‐6 promoter. ER:Ras IMR90 cells stably transfected with SIRT1 shRNA or control shRNA were given 4‐OHT for 6 days, and then, lysates were analyzed by ChIP assay using an antibody against NF‐κB. NF‐κB binding to the IL‐6 promoters in the indicated samples is represented relative to mouse immunoglobulin G binding. Error bars represent means ± SD from three independent experiments. *P <0.05.

As mentioned above, NF‐κB acetylation status during cell senescence has not been probed, and whether SIRT1 can regulate SASP by modulating NF‐κB acetylation and activity is waiting to be scrutinized. To explore this possibility, we used Ras OIS model in ER:Ras‐IMR90 cells. After Ras induction 5‐6 days, cells entered ‘senescence phase’ which characterized by elevation of p53, p21, and p16, as well as IL‐6/IL‐8 expression and secretion (Fig. 4A,B). Meanwhile, we observed a sharp decline in hnRNP A1 and SIRT1 level at day 5‐6, which was consistent with their decrease in replicative senescent cells (Fig. 4A).

We then examined the acetylation level of NF‐κB during Ras OIS and inspected whether SIRT1 could regulate NF‐κB acetylation and SASP by stably expressing SIRT1 in ER:Ras‐IMR90 cells. With Ras induction, the acetylation of p65 on K310 was gradually risen starting at day 2‐3 and reaching peak around day 4‐6 (Fig. 4C, left panel), which indicated the activation of NF‐κB during Ras OIS. Importantly, the acetylation intensity of p65 on K310 correlated well with IL‐6/IL‐8 expression, implying that the acetylation of p65 on K310 is important for NF‐κB activity and SASP induction. Conversely, SIRT1 overexpression led to deacetylate p65 on K310 during Ras OIS and significantly abolished IL‐6/IL‐8 expression (Fig. 4C, right panel).

To further verify the role of NF‐κB acetylation on SASP production, wild‐type p65 and p65 acetylation‐deficient mutant p65 (K310R) were introduced in ER:Ras‐IMR90 cells, respectively. p65 considerably augmented NF‐κB acetylation level and IL‐6/IL‐8 expression, whereas p65 (K310R) notably decreased NF‐κB acetylation and IL‐6/IL‐8 expression levels, when compared with corresponding control vector cells (Fig. 4D). Deacetylation of NF‐κB disrupts its binding activity to target DNA; we then investigated the effect of SIRT1 on the binding activity of NF‐κB to IL‐6 promoter via chromatin immunoprecipitation assay (ChIP). NF‐κB promoter binding activity to IL‐6 was drastically increased following SIRT1 deletion when compared with control cells (Fig. 4E). These results demonstrate that NF‐κB acetylation and SIRT1 deacetylating NF‐κB at K310 play important role in regulating SASP expression, and suggest that SIRT1 might prevent Ras OIS by blocking IL‐6/IL‐8 expression.

We also examined the acetylation of p65 on K310 *in vivo*. Surprisingly, the overall level of Ac‐p65 (K310) in those tissues in old mice was reduced compared with young mice (Fig. S8A–D), which was opposite to our *in vitro* data and to the reported results that NF‐κB activity is increased in multiple mammalian tissues during normal and premature aging (Adler *et al*., 2007; Tilstra *et al*., 2012; Zhang *et al*., 2013). Different cell types, senescence‐inducing stimulus, mouse strains, aged state, or animal individual variations may account for this discrepancy.

### hnRNP A1 suppresses NF‐κB acetylation and SASP via modulation of SIRT1 expression in Ras OIS

**Figure 5:**
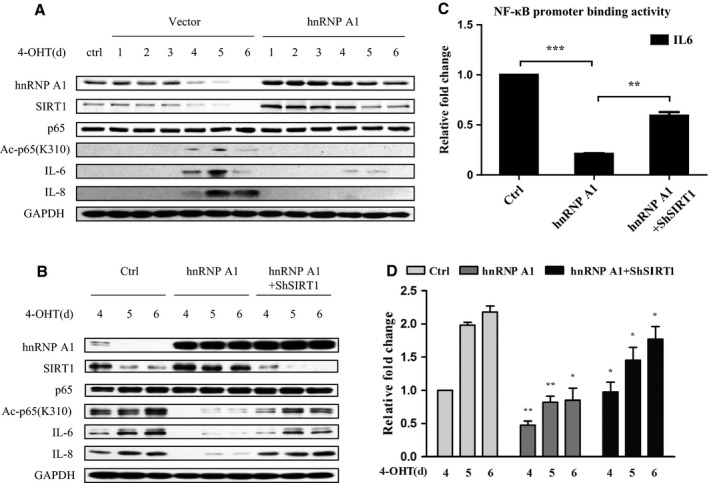
hnRNP A1 restrains NF‐κB acetylation and IL‐6/IL‐8 induction via elevation of SIRT1 expression in Ras OIS. (A) hnRNP A1 rescues SIRT1 level and represses p65 acetylation and IL‐6/IL‐8 induction. ER:Ras IMR90 cells stably transfected with hnRNP A1 or control vector were given 4‐OHT for the indicated days, and cell lysates were collected every day for total 6 days, and then followed by Western blot analysis. (B–D) hnRNP A1 suppresses p65 acetylation and IL‐6/IL‐8 expression depending on SIRT1. ER:Ras IMR90 cells stably transfected with hnRNP A1 +  ShSIRT1 or hnRNP A1 +  control vector were given 4‐OHT for the indicated days, and then cell lysates and supernatants were collected, (B) cell lysates were subjected to Western blot analysis, (C) supernatants were analyzed secretory levels of IL‐6 by ELISA, and (D) cell lysates were analyzed by ChIP assay using an antibody against NF‐κB. Error bars represent means ± SD from three independent experiments. *P <0.05, ** *P* <0.01, *** *P* <0.005.

We next explored whether hnRNP A1 could influence NF‐κB acetylation and SASP induction. To examine this, hnRNP A1 was stably transfected in IMR90 cells. hnRNP A1 overexpression restored SIRT1 level which was increasingly lost during Ras OIS (Fig. 5A). Meanwhile, the acetylation level of p65 on K310, NF‐κB promoter binding activity to IL‐6, as well as IL‐6/IL‐8 expression and secretion were notably mitigated by hnRNP A1 overexpression (Fig. 5A,C,D). These results also indicate that hnRNP A1 may avoid from Ras OIS by inhibiting NF‐κB activity and IL‐6/IL‐8 expression.

To certify whether hnRNP A1 depends on SIRT1 to regulate NF‐κB acetylation and IL‐6/IL‐8 expression, hnRNP A1 and shRNA‐SIRT1 were cotransfected in ER:Ras‐IMR90 cells. SIRT1 deletion considerably revoked hnRNP A1 ability to repress NF‐κB acetylation on K310, NF‐κB binding activity to IL‐6 promoter, and IL‐6/IL‐8 expression and secretion (Fig. 5B–D). Therefore, we conclude that hnRNP A1 relies on SIRT1 to mediate NF‐κB acetylation and IL‐6/IL‐8 expression in Ras OIS.

p38 MAPK has been reported to induce SASP by increasing NF‐κB activity (Freund *et al*., 2011). p38 also regulates NF‐κB acetylation at K310 to control its activity (Saha *et al*., 2007). Furthermore, p38 modulates hnRNP A1 level in young and G_0_‐arrested cells (Shimada *et al*., 2009). Hence, we investigate whether p38 acts as an upstream cue of hnRNP A1‐SIRT1 to regulate NF‐κB acetylation at K310 and subsequent SASP induction. To examine this, p38 activity was inhibited by adding its specific inhibitor SB203580 (SB) when Ras being induced at day 0 (young state) or day 4 (presenescent state), respectively. Repression of p38 at both day 0 and day 4 not only inhibited IL‐6/IL‐8 expression, which was consistent with previous result (Freund *et al*., 2011), but also mitigated p65 acetylation at K310, when compared with solvent treated control cells (Fig. S9A and S9B). However, compared with untreated cells, the decline in hnRNP A1 and SIRT1 level during Ras OIS was not altered by SB treatment at either day 0 or day 4.

### hnRNP A1 promotes tumor development depending on SIRT1

**Figure 6:**
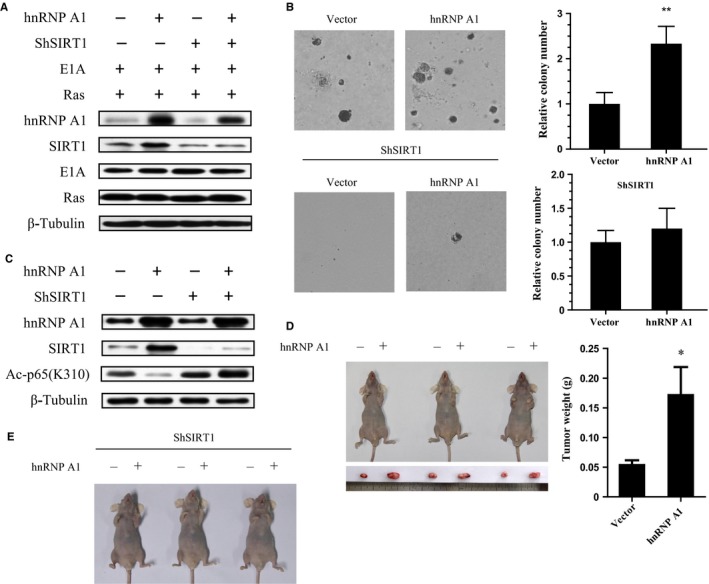
nRNP A1 enhances cell transformation and tumorigenesis in a SIRT1‐dependent manner. (A) Normal IMR90 cells were transduced with Ras, E1A, and vector, or hnRNP A1, or hnRNP A1 +  shRNA‐SIRT1. Western blot analysis was performed for the indicated proteins. (B) The stable cell lines grew in soft agar as described. Colony numbers were measured. Data are shown as the mean ± S.E., n = 3. ** *P* <0.01. (C) U2OS or U2OS‐ShSIRT1 cells were stably transfected with control vector or hnRNP A1, and indicated proteins were determined by Western blot. (D, E) Above two pairs of stable cell lines were injected into the left and right forelimb armpit of nude mice, respectively. 7 weeks after injection, tumor growth was photographed, each tumor was weighed, and its size was measured. Data are shown as the mean ± S.E., n = 3. * *P* <0.05.

hnRNP A1 overexpression led to blunt NF‐κB activity and SASP induction and bypass Ras OIS in a SIRT1‐dependent manner prompted us to explore whether hnRNP A1 overexpression would permit cell transformation in IMR90 cells. Primary human fibroblasts can be efficiently transformed by a combination of Ras, E1A, and MDM2 (Seger *et al*., 2002). We tested whether hnRNP A1 overexpression could functionally replace MDM2 expression. IMR90 cells infected with Ras, E1A, and hnRNP A1 formed robust anchorage‐independent growth colonies in soft agar, whereas Ras, E1A, and empty vector‐infected cells only formed rare and small colonies in soft agar, which agreed with previous data (Seger *et al*., 2002) (Fig. 6B). In contrast, Ras, E1A, and hnRNP A1 +  shRNA‐SIRT1‐infected cells lost the ability to form colonies in soft agar. The coexpression of Ras, E1A, hnRNP A1, and SIRT1 deletion was confirmed by Western blot (Fig. 6A).

We further examined whether hnRNP A1 could facilitate tumorigenesis via SIRT1 in immunodeficient nude mice. U2OS and U2OS‐ShRNA‐SIRT1 cells were stably transfected with hnRNP A1 or vector control, respectively (Fig. 6C), and then injected into opposite side of forelimb armpit in the same mice. Similar to our results in Ras OIS, hnRNP A1 largely alleviated p65 acetylation at K310, while SIRT1 deletion abolished this effect in U2OS cells. Nude mice inoculated with hnRNP A1‐overexpressed U2OS cells grew large tumors compared with those injected with control vector‐transfected U2OS cells (Fig. 6D), which suggested that hnRNP A1 could enhance tumor growth. However, no detectable tumors formed in either side of forelimb armpit in mice injected with U2OS‐ShRNA‐SIRT1 cells regardless of with or without hnRNP A1 overexpression (Fig. 6E). These results indicate that the ability of hnRNP A1 to promote tumor growth relies on SIRT1. Furthermore, the result also implied that SIRT1 could favor oncogenesis under certain circumstance.

## Discussion

SIRT1 plays multiple roles including CR, stress resistance, metabolism, and against age‐related diseases which tightly associated with healthy aging (Houtkooper *et al*., 2012; Giblin *et al*., 2014). However, how is SIRT1 itself regulated in aging, especially its posttranscriptional regulation is barely understood. Here, we identified that hnRNP A1 as a RNA‐binding protein directly bonds to SIRT1 mRNA 3′UTR and stabilizes SIRT1 mRNA and thus augments SIRT1 at both mRNA and protein levels (Figs 1 and 2). Therefore, our study shed new light on the posttranscriptional regulation of SIRT1 by hnRNP A1.

Further, we demonstrated that hnRNP A1‐SIRT1 played significant role in cellular senescence by suppressing p65 acetylation at K310, NF‐κB transcriptional activity, and subsequent IL‐6/IL‐8 induction. In the early phase of Ras OIS, hnRNP A1 and SIRT1 levels remain relative high and SIRT1 represses p65 acetylation by its typical deacetylase activity, and therefore, NF‐κB maintains lower transcriptional activity. However, when cells entered senescent phase, hnRNP A1 level diminished remarkably and hence attenuated SIRT1 mRNA stability and SIRT1 expression. The reduction in SIRT1 increases NF‐κB acetylation level, promotes IL‐6/IL‐8 expression and secretion, which triggers positive feedback loop between NF‐κB and SASP in autocrine pathways. Ectopic expression of SIRT1 or hnRNP A1 dampens NF‐κB acetylation, activity, and SASP induction and thus may cause the bypass of Ras OIS (Figs 4 and 5). Therefore, our findings provide the explanation for the decrease in SIRT1 during cell aging and its consequence, namely inducing SASP. It was reported that SIRT1 could directly bind to and deacetylate the histones H3 (K9) and H4 (K16) in the promoter regions of IL‐6 and IL‐8 and thus impeded their expression to negatively regulate SASP (Hayakawa *et al*., 2015). We speculate that both mechanisms are not mutually exclusive and SIRT1 can use both processes simultaneously to suppress SASP.

Although hnRNP A1 level gradually decreases during cellular senescence and hnRNP A1 depletion induces senescence phenotype (Shimada *et al*., 2009), and even assuming hnRNP A1 may exert its role in cellular senescence due to its telomere maintenance function (LaBranche *et al*., 1998; Ding *et al*., 1999), there were no direct studies to support this hypothesis. Our data show that hnRNP A1 exerts antisenescence effect independent of its telomere maintenance function by upregulating SIRT1 expression to antagonize NF‐κB activity and SASP induction.

p38 MAPK has been shown to induce SASP by increasing NF‐κB DAN‐binding activity to SASP genes (Freund *et al*., 2011). Moreover, p38 can control NF‐κB transcriptional activity via regulating p65 acetylation at K310 without altering its phosphorylation level and DNA‐binding activity (Saha *et al*., 2007). Meanwhile, inhibition of p38 activity has been reported to enhance hnRNP A1 expression in young and G_0_‐arrested cells (Shimada *et al*., 2009). Thus, it is plausible to assume that p38 may act as an upstream cue of hnRNP A1‐SIRT1 to modulate NF‐κB acetylation and subsequent SASP induction. Indeed, we observed inhibition of p38 led to reduce IL‐6/IL‐8 expression, which agreed with previous data (Freund *et al*., 2011), and decrease p65 acetylation at K310, which was not reported before (Fig. S9). These results suggest that p38 modulates SASP induction at least partly through altering p65 acetylation at K310. However, p38 inhibition had no effect on either hnRNP A1 or SIRT1 level during senescent process, which was consistent with previous result that p38 was unable to change hnRNP A1 expression level in senescent cells (Shimada *et al*., 2009). These data strongly imply that both p38 and hnRNP A1‐SIRT1 could regulate NF‐κB acetylation at K310 and SASP induction, but they exert their roles independently.

The role of SIRT1 in tumorigenesis is contradictory (Deng, 2009). Many clues point SIRT1 to a positive role in tumor development as it negatively regulates multiple tumor suppressors including p53 and forkhead proteins (Luo *et al*., 2001; Vaziri *et al*., 2001; Brunet *et al*., 2004; Motta *et al*., 2004; Deng, 2009). However, recent studies also support SIRT1 as a tumor suppressor which inhibits several oncogenic proteins such as NF‐κB, β‐catenin, and survivin under certain circumstances (Yeung *et al*., 2004; Firestein *et al*., 2008; Wang *et al*., 2008). Ras OIS is an important innate tumor suppressive mechanism and bypass of Ras OIS results in the tumor initiation and development. We demonstrate that SIRT1 expression can prevent from Ras OIS and hnRNP A1 depends on SIRT1 to promote cell transformation and oncogenesis. These results support the notion that SIRT1 functions as a potential oncogene at least in the context of oncogenic Ras activation.

Considering the upregulation of hnRNP A1 in various tumors and its roles in cancer development by promoting tumor invasion and so on (Pino *et al*., 2003; Ushigome *et al*., 2005; Carpenter *et al*., 2006; Boukakis *et al*., 2010; Zhou *et al*., 2013), hnRNP A1 caused escaping from Ras OIS and promoting cell transformation and tumor development in nude mice in SIRT1‐dependent manner provides novel explanation for its role in tumorigenesis. NF‐κB activation and subsequent SASP induction have been observed in some type of drug‐induced cancer cell senescence (DIS) treated by certain antitumor agents and notably contribute to patient outcome after cancer chemotherapy by stimulating immune system to eliminate senescent cancer cells. Diminishing NF‐κB activity and subsequent SASP expression largely reduces the clearance of senescent cancer cells and causes them to accumulate *in vivo* and eventually leads to cancer relapse (Chien *et al*., 2011; Jing *et al*., 2011). It was reported that hnRNP A1 upregulation in hepatocellular carcinoma resulted in poor prognosis (Zhou *et al*., 2013). The ability of hnRNP A1 to inhibit NF‐κB activity and SASP induction we show here may suggest a novel mechanism to elucidate hnRNP A1 attribution to poor prognosis in cancer treatment. Therefore, hnRNP A1 and SIRT1 may represent potential diagnostic and therapeutic targets for certain cancer treatments.

In summary, we report here the novel posttranscriptional regulation of SIRT1 by hnRNP A1 which binds to SIRT1 mRNA 3′UTR to stabilize SIRT1 mRNA and increase SIRT1 expression. We also demonstrate that hnRNP A1‐SIRT1 axis delays replicative cell senescence and prevents from Ras OIS by abrogation of NF‐κB acetylation and activity and subsequent IL‐6/IL‐8 induction.

## Experimental procedures

Antibodies, reagents, and cell lines used in this study are described in Supplemental Experimental Procedures. All of experiments were processed according to the standard protocols. Plasmids, viral infection, real‐time PCR, ChIP, immunoblot analysis, RNP‐IP, biotinylated RNA pull‐down assay, luciferase reporter gene assays, RNA isolation, RNA half‐life determination, SA‐β‐gal staining, cell cycle analysis, ELISA, soft agar, tumorigenic assay, etc., are provided in detail in Supplemental Experimental Procedures.

## Funding

This work was supported by National Key Basic Research Program of China Grants 2013CB530801 and 2012CB911203.

## Conflict of interest

All the authors declare no conflict of interest.

## Author contributions

HW and JC designed research; HW, LMH, GYZ, HS, PFW, ZMS, CZX, YYS, and GDL performed research and analyzed the data; TJT supervised the research; and HW and JC wrote the manuscript.

## Supporting information


**Fig. S1** Identify the specific binding region on SIRT1 mRNA 3′UTR to hnRNP A1 in H1299 cells
**Fig. S2** Statistical analysisof hnRNP A1 and SIRT1 expression
**Fig. S3** hnRNP A1 doesn't alter SIRT1 pre‐RNA level
**Fig. S4** The interaction between three different fragments of SIRT1 mRNA 3′UTR withhnRNP A1
**Fig. S5** Statistical analysisof hnRNP A1 and SIRT1 expression
**Fig. S6** hnRNP A1 delays replicative cellular senescence dependent onSIRT1in 2BS cells
**Fig. S7** shRNA‐hnRNP A1‐2 induces senescence phenotypeand shRNA‐SIRT1‐2 counteracts hnRNP A1 effect ofdelaying cellular senescence
**Fig. S8** hnRNP A1 and SIRT1 levels decrease in multiple tissues in old mice
**Fig. S9** p38 MAPK regulates p65 acetylation at K310 and IL‐6/IL‐8 induction during Ras OIS independent of hnRNP A1‐SIRT1.

**Table S1** related to Figure 1: Mass Spectrometry Analysis of SIRT1 mRNA binding proteins.
