# Supplementary material for: hnRNP A1 antagonizes cellular senescence and senescence‐associated secretory phenotype via regulation of SIRT1 mRNA stability
Source: Aging Cell. 2016 Sep 9;15(6):1063–73. doi: 10.1111/acel.12511 (PMC6398525; doi:10.1111/acel.12511)
Supplement: Supplementary file 1 — Fig. S1 Identify the specific binding region on SIRT1 mRNA 3′UTR to hnRNP A1 in H1299 cells Fig. S2 Statistical analysisof hnRNP A1 and SIRT1 expression Fig. S3 hnRNP A1 doesn't alter SIRT1 pre‐RNA level Fig. S4 The interaction between three different fragments of SIRT1 mRNA 3′UTR withhnRNP A1 Fig. S5 Statistical analysisof hnRNP A1 and SIRT1 expression Fig. S6 hnRNP A1 delays replicative cellular senescence dependent onSIRT1in 2BS cells Fig. S7 shRNA‐hnRNP A1‐2 induces senescence phenotypeand shRNA‐SIRT1‐2 counteracts hnRNP A1 effect ofdelaying cellular senescence Fig. S8 hnRNP A1 and SIRT1 levels decrease in multiple tissues in old mice Fig. S9 p38 MAPK regulates p65 acetylation at K310 and IL‐6/IL‐8 induction during Ras OIS independent of hnRNP A1‐SIRT1. [file ACEL-15-1063-s001.pdf]

Supplemental Experimental Procedures

Cell culture, Reagents, Transfection, and RNA Interference

Human embryonic lung diploid fibroblast 2BS cells (National Institute of Biological Products, Beijing, China) and human cell lines HEK293, HEK293T, HeLa, U2OS, H1299 were from our laboratory and cultured in Dulbecco's modified Eagle's medium (DMEM) supplemented with 10% fetal bovine serum (FBS) and maintained in a humidified incubator equilibrated with 5% CO2 at 37 ℃. Cells were transiently transfected with plasmids using Lipofectamine 2000 Reagent (Invitrogen) and siRNAs were transfected using Lipofectamine RNAiMAX Reagent following the manufacturer's protocol. 48 h or 72 h after transfection, cells were harvested and lysed to evaluate the transfection efficiency. Two independent hnRNP A1 target siRNA sequences and the lentivirus vector pLL3.7-shhnRNP A1 sequences were: 5′- AAU GAG AGA UCC AAA CAC CAA -3′ and 5' -CAA CUU CGG UCG UGG AGG A -3'. Two independent siRNA sequences of SIRT1 and the oligonucleotides corresponding to shRNA targeting SIRT1 mRNA sequence were: 5′- ACU UUG CUG UAA CCC UGU A -3′ and 5′- GAA GUU GAC CUC CUC AUU GU -3′.

Cell-Cycle Analysis

Cells with different treatments were washed three times with PBS, detached with 0.25% Trypsin, and fixed with 75% ethonal at -20 ℃ overnight. After treatment with 2.5μl 10 mg/ml RNase A (Fermentas) at 37 ℃ for 30 min, cells were resuspended in 500μl of PBS and stained with propidium iodide in the dark for 30 min. Cells were filtered and fluorescence was measured with a FACScan flow cytometry system (BD Biosciences).

SA-β-Gal activity and Colony Formation

For SA-β-gal staining, cells were washed twice with ice-cold 1×PBS, fixed for 10 min at room temperature in 3% formaldehyde, and washed twice with 1× PBS. Then cells were incubated in a freshly prepared SA-β-gal staining solution at 37 ℃ without CO_2 overnight followed by photomicrography.

To perform colony formation, 1×10^3, 3×10^3, and 1×10^4 cells were cultured in six-well plate. Several days later, cells were fixed in 3% formaldehyde at 37 ℃ for 30 min and washed twice with 1 ×PBS, then stained with crystal violet for 1 h and washed with 1 ×PBS twice followed by photography.

Cell Growth Curves

Cells stably transfected with sh-hnRNP A1 or sh-control were seeded into 6-well plate at a density 1×10^4 cells per well and cultured for periods ranging from 1 to 7 d. The medium was changed every 24 h. At the indicated times, the confluence of cells were determined using CloneSelect Imager system (Genetix). Values are the mean ±S.D. of six points from a representative experiment and repeated more than three times with similar results.

RNA isolation, Real-time PCR and half-life determination

Total cellular RNA was isolated using the RNeasy Mini kit (Qiagen) following the manufacturer's protocol. Then the cDNA was synthesized using TransScript First-Strand cDNA Synthesis SuperMix (Transgen) followed by real-time PCR analysis with SYBR Select Master Mix (Applied Biosystems) on an ABI PRISM 7500 Sequence Detector (Applied Biosystems) with the

expression of GAPDH as the internal control. The sequences of the primers used are provided:

hnRNP A1 forward： 5′- CTAAAGAGCCCGAACAGCTGAG -3′

and reverse: 5′-TCAGTGTCTTCTTTAATGCCACCA-3′

SIRT1 forward：5'-CTGATGAACCGCTTGCTAT-3'

and reverse: 5'-CTACTGGTCTTACTTTGAGGGA-3'

p16 forward: 5'-GCCCAACGCACCGAATAGT-3'

and reverse: 5'-CGCTGCCCATCATCATGAC-3'

GAPDH forward: 5'-CGACCACTTTGTCAAGCTCA-3'

and reverse: 5'-AGGGGTCTACATGGCAACTG-3'

To measure the half-life of SIRT1 mRNA, the expression of SIRT1 mRNA was shut off by adding actinomycin D (2 μg/ml) into the cell culture medium, and total RNA was prepared at the times indicated and subjected to RT-qPCR analysis using SIRT1-specific primers.

Western Blot

Cells were collected in RIPA buffer with protease inhibitor (Cocktails, AMRESCO), and lysed on ice for 30 min with short vortex per 10 min. Lysates were centrifuged for 15 min at 13,000 × g at 4 ℃ and supernatants were collected and protein concentrations were determined by BCA Protein Assay Reagent (Pierce). Lysates were size-fractionated by SDS-PAGE and transferred onto NC membranes. For western blotting analysis, membranes were incubated with primary antibodies for overnight at 4 ℃ followed by incubation with a secondary antibody for 1h at r.t. Then the signals were detected by enhanced chemiluminescence or fluorescence according to the manufacturer’s recommendation.

RNP IP

For UV cross-link RNP IP assays, cells were exposed to UVC (400 mJ/cm^2) and total RNA extracts from HeLa cells were prepared for immunoprecipitation using monoclonal anti-hnRNP A1 and IgG. The RNP were washed three times with stringent buffer (100 mM Tris-HCl, pH 7.4, 500 mM LiCl, 0.1% Triton X-100, 1 mM DTT, 2 μg/ml leupeptin, 2 μg/ml aprotinin, 1 mM phenylmethylsulfonyl fluoride) and twice with IP buffer (22). The mRNA in RNP complex was then isolated by standard procedures and analyzed by real-time PCR.

Biotinylated RNA pull down assay

cDNA was used as a template for PCR amplification of the different fragments of SIRT1 mRNA. All 5’ primers contained the T7 promoter sequence: 5’-CCAAGCTTCTAATACGACTCACTATAGGGAGA-3’ (T7). To prepare templates for 5’UTR (positions 1 to 53), and CR (coding region; positions 54 to 2297), as well as 3’UTR (positions 2298 to 4110), fragments 3’UTR-1 (positions 2298 to 2494), 3’UTR-2 (positions 2476 to 2703), 3’UTR-3 (positions 2685 to 2912), 3’UTR-4 (positions 2874 to 3101), 3’UTR-5 (positions 3083 to 3301), 3’UTR-6 (positions 3282 to 3417), 3’UTR-7 (positions 3400 to 3738), 3’UTR-8 (positions 3718 to 3862), 3’UTR-9 (positions 3845 to 4093), 3’UTR-A (positions from 2298 to 2396), 3’UTR-B (positions from 2397 to 2494), 3’UTR-C (positions from 2298 to 2363), 3’UTR-D (positions from 2364 to 2429), 3’UTR-E (positions from 2430 to 2494), 3’UTR-1 M1, M2 and M3, the following primer pairs were used: (T7)GTCGAGCGGGAGCAGAGGA and GCCATCTTCCAACTGCCTCTCTG for 5’UTR, (T7)AGTTGGAAGATGGCGGACGAG and

CACTATGATTTGTTTGATGG for CR,
(T7) TGTAATAATTGTGCAGGTACAGG and AAGTTAACAGAAAAAAGTC for 3'UTR, (T7)TGTAATAATTGTGCAGGTACAGG and GTTAGTGTTGAGTTTGTACAAG for 3'UTR-1, M1, M2 and M3, (T7)GTACAAACTCAACACTAAC and GGTCTAGATTAGCTGTTCC for 3'UTR-2, (T7)GGAACAGCTAATCTAGACC and AAGACAACCGAGTGCTCTC for 3'UTR-3, (T7)GGAAGTCAACAATATGTGG and ATCCAGGCAGGCACTAATGTT for 3'UTR-4, (T7)CATTAGTGCCTGCCTGGAT and GCCTGTTGCTCTCCTCAT for 3'UTR-5, (T7)TAATGAGGAGAGCAACAGGC and CATAATACTAGGGCTAGCAG for 3'UTR-6, (T7)GCTAGCCCTAGTATTATGGAG and CTGGCAGTAATGGTCCTAGCT for 3'UTR-7, (T7)AGCTAGGACCATTACTGCCA and CTATAGCACACAAACATCATGC for 3'UTR-8, (T7)GATGTTTGTGTGCTATAGATG and AAGTTAACAGAAAAAAGTC for 3'UTR-9, (T7)TGTAATAATTGTGCAGGTACAGG and GTTTCCTTGCTCTATCGAGTTCAC for 3'UTR-A, (T7) CAGAAAGGTGTAATATTTATAGG and GTTAGTGTTGAGTTTGTACAAGT for 3'UTR-B, (T7)TGTAATAATTGTGCAGGTACAGG and TTCATTTTGACATGCTAAAGTTCC for 3'UTR-C, (T7)TGTTTACTTGTGAACTCGATAGAGC and ATTTTACCAACCTATAAATATTAC for 3'UTR-D, (T7)AGATTGTTTTTCATGGATAATTTTT and GTTAGTGTTGAGTTTGTACAAGT for 3'UTR-E.

Biotinylated RNA probes were prepared using *in vitro* transcription of PCR-amplified DNA templates with T7 RNA polymerases (Promega) in the presence of the biotin-UTP labeling NTP mixture (Promega) as recommended. The reactions were incubated for 2-4 h at 37°C, followed by incubation with DNaseI (Transgene; 1 U/1 μg of template DNA) for 30 min at 37°C, and terminated by 10 mM EDTA with incubation at 65 ℃ for 10min. The biotinylated RNAs were then extracted with phenol-chloroform (1:1) mixture, precipitated with ethanol and rehydrated in DEPC-treated water. 500 nanograms of purified biotinylated transcripts were incubated with 100 μg of total cell lysates for 30 min at room temperature with continuous rotation. Complexes were isolated with streptavidin-conjugated Dynabeads (Invitrogen), followed by boiling with SDS–PAGE loading buffer for 5 min. The pulldown materials were subsequently analyzed by Western blotting by probing the membranes successively with hnRNP A1-specific and β-Tubulin-specific antibody.

Luciferase reporter gene assays

For the construction of pGL3-derived reporter vectors bearing 5'UTR, CR, 3'UTR and different fragments of SIRT1 mRNA with XbaI or XmaJI (Isocandamer of XbaI) restriction enzyme cutting site, the following primer pairs were used :

5'UTR forward: 5'-GCTCTAGAGTCGAGCGGGAGCAGAGGA-3'
reverse: 5'-GCTCTAGAGCCATCTTCCAACTGCCTCTCTG-3'
CR forward: 5'-GCTCTAGAATGGCGGACGAGGCGGCCCT-3'
reverse: 5'- GCTCTAGACTATGATTTGTTTGATGG-3'
3'UTR forward: 5′- CCTAGGTGTAATAATTGTGCAGGTA-3'
reverse: 5'- CCTAGG AAGTTAACAGAAAAAAGTC-3'
3'UTR-1 forward: 5′- GCTCTAGATGTAATAATTGTGCAGGTACAGG-3'
reverse: 5'- GCTCTAGAAAGACAACCGAGTGCTCTC-3'

3’UTR-2 forward: 5′-GCTCTAGAGGAAGTCAACAATATGTGG-3’
reverse: 5’- GCTCTAGACATAATACTAGGGCTAGCAG-3’
3’UTR-3 forward: 5′-GCTCTAGAGCTAGCCCTAGTATTATGGAG-3’
reverse: 5’- GCTCTAGAAAGTTAACAGAAAAAAGTC-3’

For reporter gene assays, the constructed luciferase-reporter vectors and Renilla vectors as loading control were transfected using Lipofectamine 2000 (Invitrogen) following the manufacturer’s instructions. Cell lysates were collected, and the luciferase activities against Renilla luciferase activities were measured with the double-luciferase assay system (Promega) following the manufacturer’s instructions.

ELISA

The supernatants of IMR90 cells were collected after treatment as indicated. Human IL-6 level was measured by an ELISA kit from Boster.

Soft Agar Assay

The transformation cells and control cells were cultured in 0.4% agar on a cushion of 0.6% agar in six-cm dish for 14 d, then the colonies were observed under a light microscope.

Tumorigenicity in Nude Mice

U2OS or ShSIRT1-U2OS cells were stably transfected with either control vector or hnRNP A1 overexpression vector. After selection, 3×10^6 cells were suspended in 200 μl of PBS and subcutaneously injected into the left and right forelimb armpit of 4-week-old male nude mice, respectively. 7 weeks after injection, the mice were killed, the tumors were weighed, and tumor sizes were measured. Each sub-cell line was evaluated in three animals.

Statistical analysis

Results are depicted as mean values ± standard deviation (SD). The data are presented as x-fold induction compared with untreated conditions (*). Statistical analysis was performed using Student’s *t*-test. $P < 0.05$ (*), or $P < 0.01$ (**), or $P < 0.005$ (***) were considered significant.

Supplemental Figure Legends

Fig. S1 Identify the specific binding region on SIRT1 mRNA 3'UTR to hnRNP A1 in H1299 cells (related to Fig. 1E)

9 different fragments of SIRT1 mRNA 3'UTR were biotinylated and detected by Western blotting. CR of SIRT1 mRNA was considered as negative RNA probe to hnRNP A1. 5μg aliquot of whole cell lysate was included as input, RNA binding protein TIA-1 and β-Tubulin were blotted as negative controls.

Fig. S2 Statistical analysis of hnRNPA1 and SIRT1 expression (related to Fig. 2A)

hnRNP A1 overexpression enhances SIRT1 expression, whereas hnRNP A1 silencing decreases SIRT1 induction. Results are depicted as mean values ±standard deviation (SD). The data are presented as x-fold induction compared with untreated conditions (*). Statistical analysis was performed using Student's *t*-test. $P < 0.05$ (*), or $P < 0.01$ (**), or $P < 0.005$ (***) were considered significant.

Fig. S3 hnRNP A1 doesn't alter SIRT1 pre-RNA level

H1299 cells were transiently transfected with hnRNP A1-pcDNA3.1 and pcDNA3.1 vector, or hnRNP A1 siRNA and control siRNA. After 72 h transfection, cells were harvested, total RNA were extracted and subjected to RT-qPCR analysis to evaluate the mRNA and pre-RNA levels of SIRT1. hnRNP A1 mRNA level served as a positive control. Error bars represented as means ± SD from three independent experiments.

Fig. S4 The interaction between three different fragments of SIRT1 mRNA 3'UTR with hnRNP A1 (related to Fig. 2E)

(A) Schematic representation of SIRT1 mRNA, and SIRT1 mRNA 3'UTR was separated into three different fragments, in accordance with Luciferase reporter gene assays (Fig. 2E).

(B) Three fragments, 3'UTR-1 and 3'UTR-2 and 3'UTR-3 of SIRT1 3'UTR were biotinylated and detected by RNA pull down following Western Blotting. 5'UTR and CR of SIRT1 mRNA were considered as negative RNA probes to hnRNP A1. 5μg aliquot of whole cell lysate was included as input, and β-Tubulin served as a negative control.

Fig. S5 Statistical analysis of hnRNPA1 and SIRT1 expression (related to Fig. 3C)

Stable transfection of hnRNP A1 or Sh-hnRNP A1 in 2BS cells. Results are depicted as mean values ±standard deviation (SD). The data are presented as x-fold induction compared with untreated conditions (*). Statistical analysis was performed using Student's *t*-test. $P < 0.05$ (*), or $P < 0.01$ (**), or $P < 0.005$ (***) were considered significant.

Fig. S6 hnRNP A1 delays replicative cellular senescence dependent on SIRT1 in 2BS cells

(A) All stable transfected cells were stained for SA-β-gal. Young and senescent 2BS cells with no treatment were also stained as controls (related to Fig. 3D).

(B) Cells were stained by DAPI to detect SAHF formation (related to Fig. 3E).

(C) Statistical analysis of hnRNP A1 and SIRT1 expression (related to Fig. 3I).

(D) All stable transfected cells were stained for SA-β-gal (related to Fig. 3I).

(E) hnRNP A1 doesn't change p53/p21, p16, and PTEN/p27 expression**.** H1299 cells were

transiently transfected with hnRNP A1-pcDNA3.1 and pcDNA3.1 vector, or hnRNP A1 siRNA and control siRNA. After 48 hours transfection, whole-cell lysates were prepared and subjected to SDS-PAGE to evaluate p53/p21, p16, and PTEN/p27 expression levels. β-Tubulin served as a loading control.

Fig. S7 shRNA-hnRNP A1-2 induces senescence phenotype and shRNA-SIRT1-2 counteracts hnRNP A1 effect of delaying cellular senescence

(A) Two distinct hnRNP A1 siRNA oligos (1 and 2) were transiently tranfected into H1299 cells. After 72h transfection, whole-cell lysates were prepared and subjected to SDS-PAGE to evaluate the efficiency of two siRNA oligos. β-Tubulin served as a loading control (related to Fig. 3C).

(B, C) Sh-hnRNP A1-2 and control vector were stably transfected in 2BS cells and cultured consistent to senescence, then stained for SA-β-gal (related to Fig. 3C and 3D). Three independent experiments were analyzed. Error bars represent means + SD (n = 5).

(D, E) hnRNP A1 regulates cell senescence dependent on SIRT1. Sh-SIRT1-2 and correspondent vectors were stably transfected in 2BS cells. Each stable cells were subjected to SA-β-gal staining after continuous passage to senescence phenotype displayed (related to Fig. 3I).

Fig. S8 hnRNP A1 and SIRT1 levels decrease in multiple tissues in old mice

(A-D) Total tissue lysates of liver, fat, heart, and muscle from four young (Y) and four old (O) mice were extracted and subjected to western blot analysis for indicated proteins.

Fig. S9 p38 MAPK regulates p65 acetylation at K310 and IL-6/IL-8 induction during Ras OIS independent of hnRNP A1-SIRT1

(A) Early inhibition of p38 abolishes p65 acetylation and IL-6/IL-8 induction, but does not alter hnRNP A1 and SIRT1 levels. ER:Ras IMR90 cells were treated with solvent DMSO or p38 kinases inhibitor SB203580 (10 μM) in the presence of 4-OHT for the indicated days, then analyzed for expression of indicated proteins.

(B) Late inhibition of p38 represses p65 acetylation and IL-6/IL-8 induction, but has no effect on hnRNP A1 and SIRT1 levels. ER:Ras IMR90 cells were induced to express Ras for 4 d, then solvent DMSO or p38 kinases inhibitor SB203580 (10 μM) were added for another 1 or 2 d in the presence of 4-OHT. The indicated proteins were analyzed by Western blot.

Supplemental Figures

Fig. S1

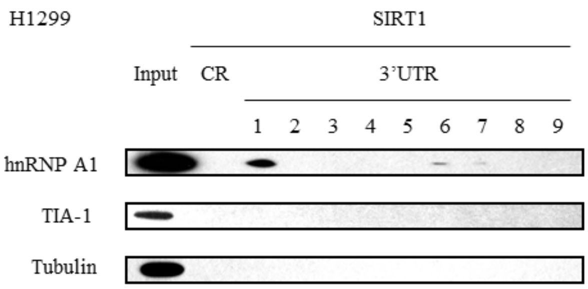

Fig. S2

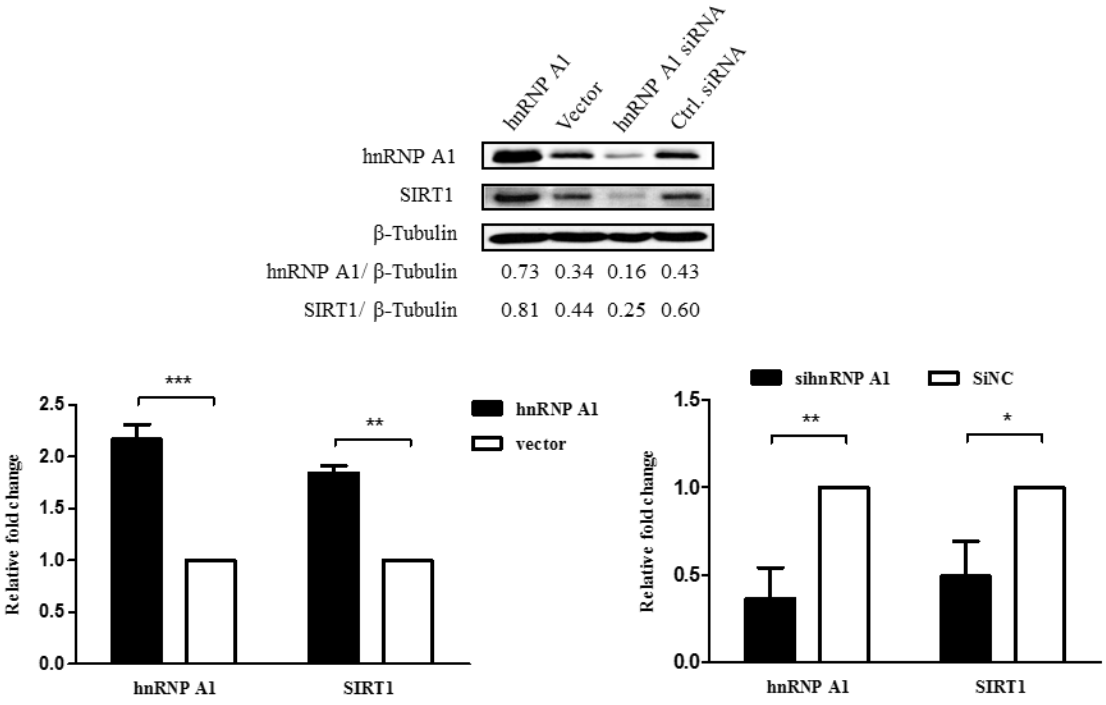

hnRNP A1
Vector
hnRNP A1 siRNA
Ctrl. siRNA
hnRNP A1
SIRT1
β-Tubulin
hnRNP A1/ β-Tubulin 0.73 0.34 0.16 0.43
SIRT1/ β-Tubulin 0.81 0.44 0.25 0.60
Relative fold change
***
**
hnRNP A1
vector
hnRNP A1
SIRT1
sihnRNP A1
SiNC
*

Fig. S3

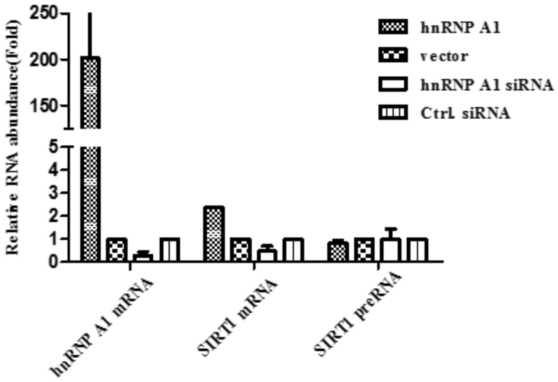

Relative RNA abundance(Fold)
250
200
150
5
4
3
2
1
0
hnRNP A1
vector
hnRNP A1 siRNA
Ctrl. siRNA
hnRNP A1 mRNA
SIRT1 mRNA
SIRT1 preRNA

Fig. S4

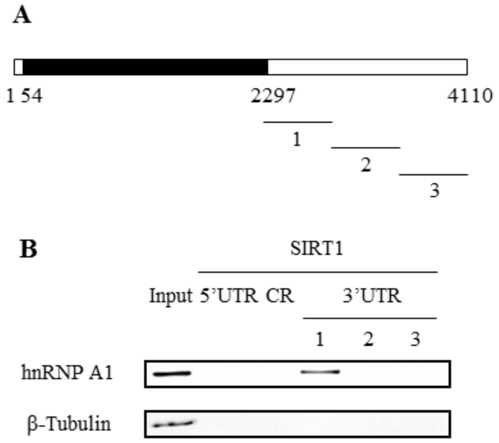
A
1 54
2297
4110
1
2
3
B
SIRT1
Input 5'UTR CR
3'UTR
1 2 3
hnRNP A1
β-Tubulin

Fig. S5

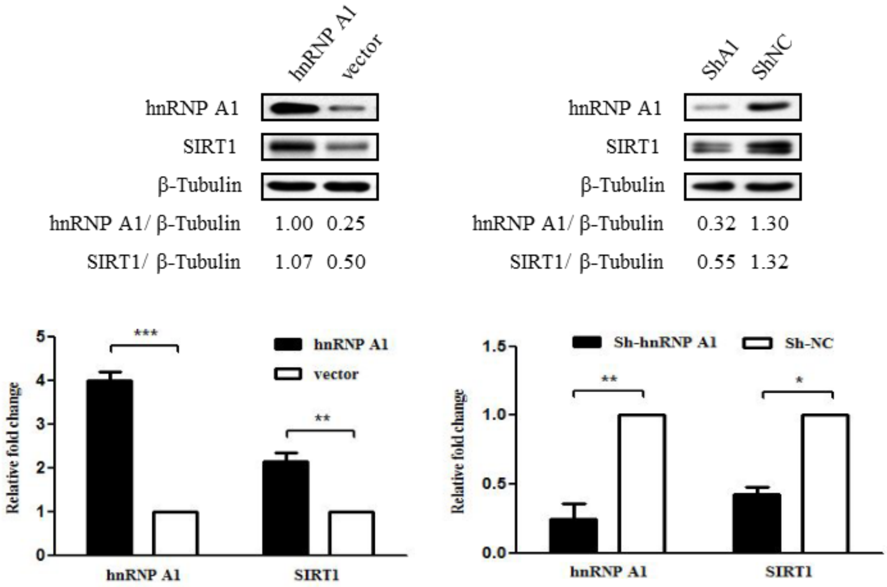
hnRNP A1
vector
ShA1
ShNC
hnRNP A1
SIRT1
β-Tubulin
hnRNP A1/ β-Tubulin 1.00 0.25
SIRT1/ β-Tubulin 1.07 0.50
hnRNP A1/ β-Tubulin 0.32 1.30
SIRT1/ β-Tubulin 0.55 1.32
Relative fold change
hnRNP A1
vector
Sh-hnRNP A1
Sh-NC
***
**
*
hnRNP A1
SIRT1
0
1
2
3
4
5
0.0
0.5
1.0
1.5

Fig. S6

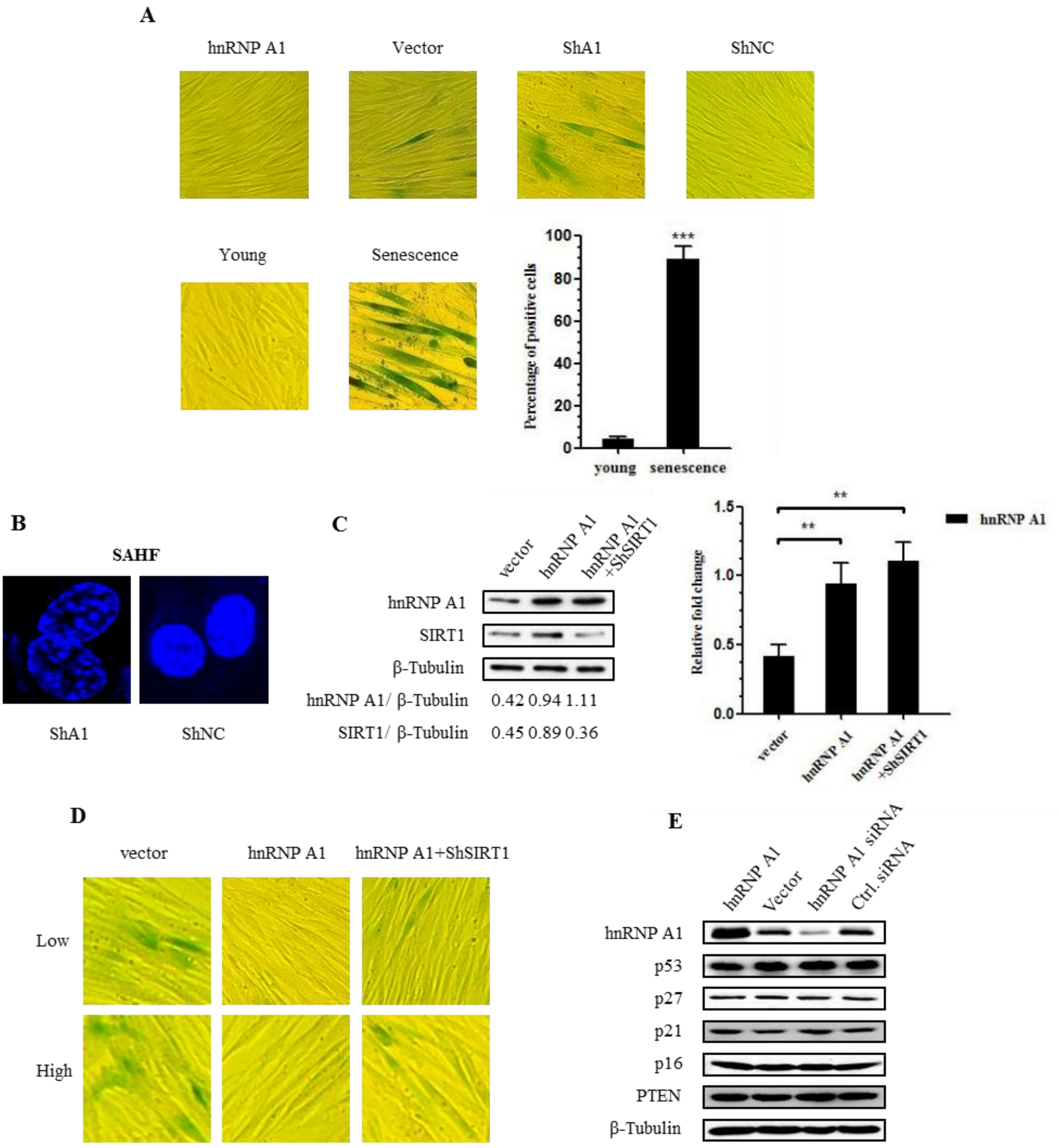
A
hnRNP A1
Vector
ShA1
ShNC
Young
Senescence
Percentage of positive cells
***
young
senescence
B
SAHF
ShA1
ShNC
C
vector
hnRNP A1
hnRNP A1 +ShSIRT1
hnRNP A1
SIRT1
β-Tubulin
hnRNP A1/ β-Tubulin 0.42 0.94 1.11
SIRT1/ β-Tubulin 0.45 0.89 0.36
Relative fold change
hnRNP A1
D
vector
hnRNP A1
hnRNP A1+ShSIRT1
Low
High
E
hnRNP A1
Vector
hnRNP A1 siRNA
Ctrl. siRNA
hnRNP A1
p53
p27
p21
p16
PTEN
β-Tubulin

Fig. S7

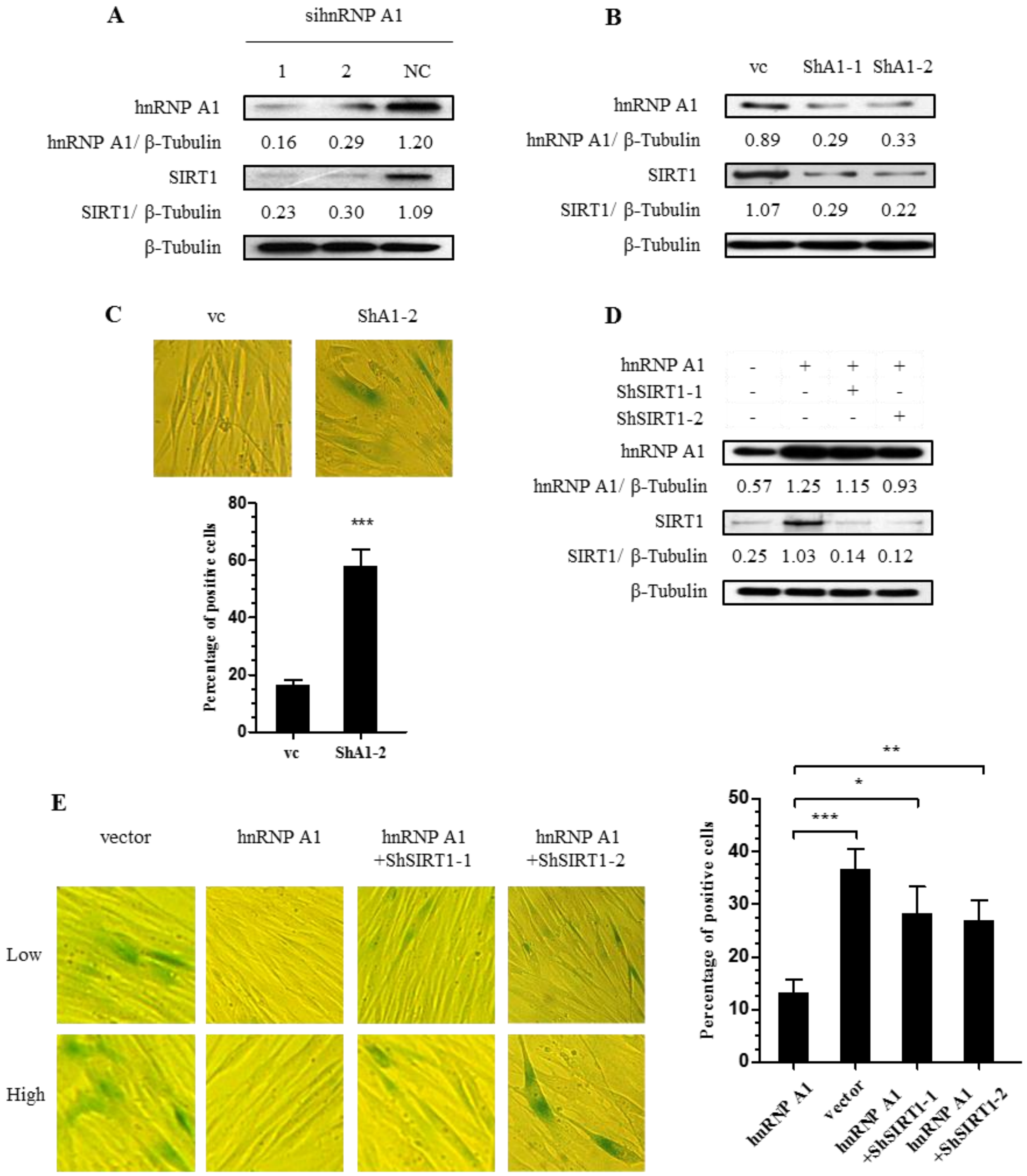
A
sihnRNP A1
1
2
NC
hnRNP A1
hnRNP A1/ β-Tubulin
0.16
0.29
1.20
SIRT1
SIRT1/ β-Tubulin
0.23
0.30
1.09
β-Tubulin
B
vc
ShA1-1
ShA1-2
hnRNP A1
hnRNP A1/ β-Tubulin
0.89
0.29
0.33
SIRT1
SIRT1/ β-Tubulin
1.07
0.29
0.22
β-Tubulin
C
vc
ShA1-2
Percentage of positive cells
***
vc
ShA1-2
D
hnRNP A1 - + + +
ShSIRT1-1 - - + -
ShSIRT1-2 - - - +
hnRNP A1
hnRNP A1/ β-Tubulin 0.57 1.25 1.15 0.93
SIRT1
SIRT1/ β-Tubulin 0.25 1.03 0.14 0.12
β-Tubulin
E
vector
hnRNP A1
hnRNP A1 +ShSIRT1-1
hnRNP A1 +ShSIRT1-2
Low
High
Percentage of positive cells
***
*
**
hnRNP A1
vector
hnRNP A1 +ShSIRT1-1
hnRNP A1 +ShSIRT1-2

Fig. S8

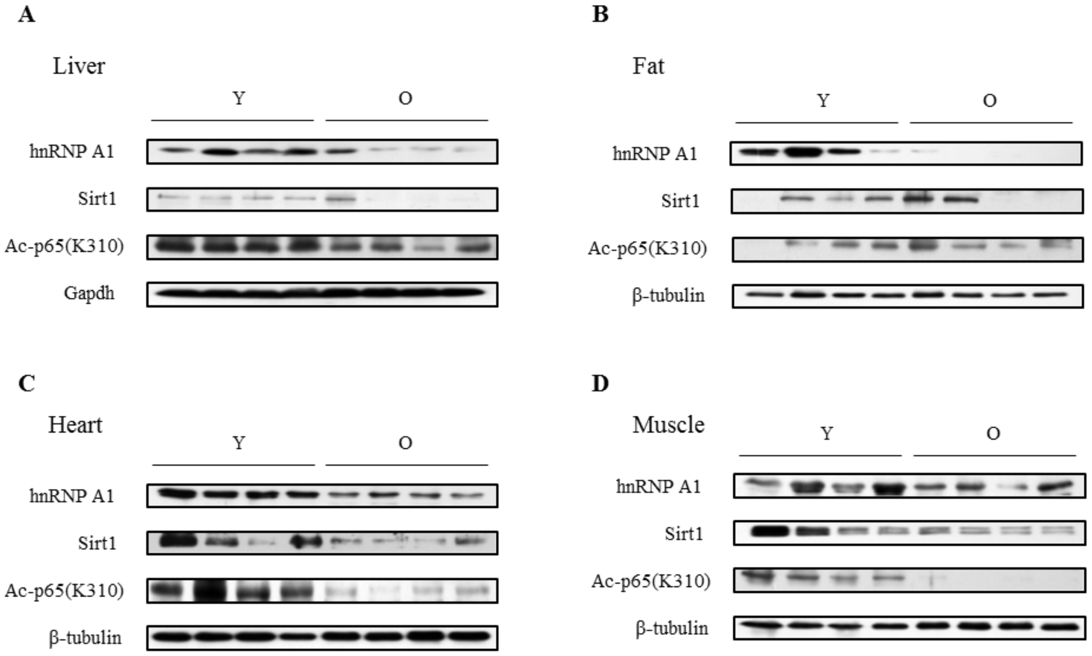
A
Liver
Y
O
hnRNP A1
Sirt1
Ac-p65(K310)
Gapdh
B
Fat
Y
O
hnRNP A1
Sirt1
Ac-p65(K310)
β-tubulin
C
Heart
Y
O
hnRNP A1
Sirt1
Ac-p65(K310)
β-tubulin
D
Muscle
Y
O
hnRNP A1
Sirt1
Ac-p65(K310)
β-tubulin

Fig. S9

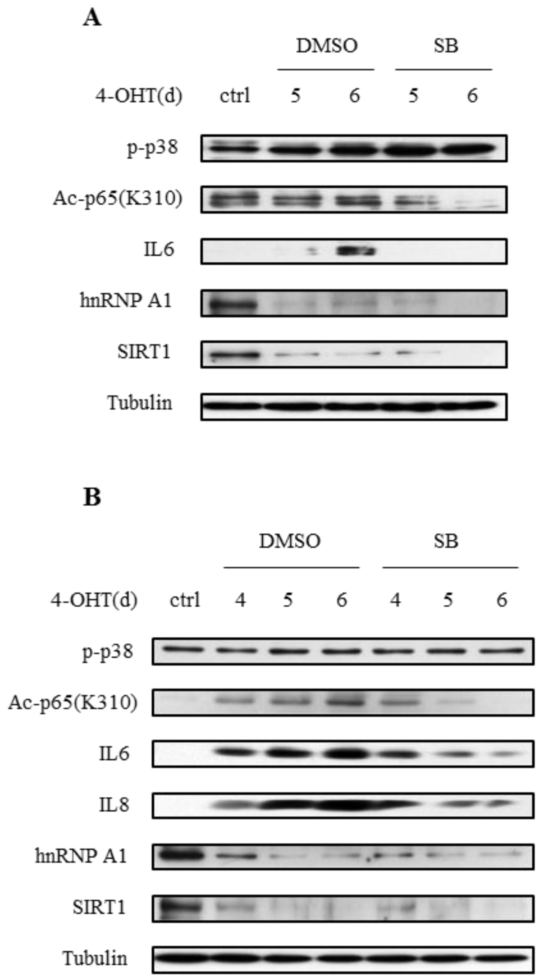
A
DMSO
SB
4-OHT(d)
ctrl
5
6
5
6
p-p38
Ac-p65(K310)
IL6
hnRNP A1
SIRT1
Tubulin
B
DMSO
SB
4-OHT(d)
ctrl
4
5
6
4
5
6
p-p38
Ac-p65(K310)
IL6
IL8
hnRNP A1
SIRT1
Tubulin
